# Aging and Cerebral Glucose Metabolism: ^18^F-FDG-PET/CT Reveals Distinct Global and Regional Metabolic Changes in Healthy Patients

**DOI:** 10.3390/life13102044

**Published:** 2023-10-12

**Authors:** Robert Christopher Subtirelu, Eric Michael Teichner, Yvonne Su, Omar Al-Daoud, Milan Patel, Shiv Patil, Milo Writer, Thomas Werner, Mona-Elisabeth Revheim, Poul Flemming Høilund-Carlsen, Abass Alavi

**Affiliations:** 1 Department of Radiology, Hospital of the University of Pennsylvania, Philadelphia, PA 19104, USA; 2 Sidney Kimmel Medical College, Thomas Jefferson University, Philadelphia, PA 19144, USA; 3 The Intervention Center, Division of Technology and Innovation, Oslo University Hospital, Sognsvannsveien 20, 0372 Oslo, Norway; 4 Institute of Clinical Medicine, Faculty of Medicine, University of Oslo, Problemveien 7, 0315 Oslo, Norway; 5 Department of Nuclear Medicine, Odense University Hospital, 5000 Odense, Denmark; 6 Department of Clinical Research, University of Southern Denmark, 5230 Odense, Denmark

**Keywords:** aging, PET/CT, 18F-FDG, glucose metabolism, quantitative analysis

## Abstract

Alterations in cerebral glucose metabolism can be indicative of both normal and pathological aging processes. In this retrospective study, we evaluated global and regional neurological glucose metabolism in 73 healthy individuals (mean age: 35.8 ± 13.1 years; 82.5% female) using 18F-Fluorodeoxyglucose (FDG) positron emission tomography/computed tomography (PET/CT). This population exhibited a low prevalence of comorbidities associated with cerebrovascular risk factors. We utilized ^18^F-FDG-PET/CT imaging and quantitative regional analysis to assess cerebral glucose metabolism. A statistically significant negative correlation was found between age and the global standardized uptake value mean (SUVmean) of FDG uptake (*p* = 0.000795), indicating a decrease in whole-brain glucose metabolism with aging. Furthermore, region-specific analysis identified significant correlations in four cerebral regions, with positive correlations in the basis pontis, cerebellar hemisphere, and cerebellum and a negative correlation in the lateral orbital gyrus. These results were further confirmed via linear regression analysis. Our findings reveal a nuanced understanding of how aging affects glucose metabolism in the brain, providing insight into normal neurology. The study underscores the utility of ^18^F-FDG-PET/CT as a sensitive tool in monitoring these metabolic changes, highlighting its potential for the early detection of neurological diseases and disorders related to aging.

## 1. Introduction

Aging is a physiological process that affects all organs, including the brain and nervous system. The degree of decline associated with healthy versus pathological aging depends on the severity of the observed changes [1]. A thorough understanding of both healthy and pathological aging across all organ systems can contribute to the establishment of better individualized care for patients. Aging of the nervous system can be identified according to alterations in the neurological exam, including changes in sensory perception reflexes, visual and auditory functions, taste, smell, motor coordination, movement abnormalities, and cognition [2,3]. Neurological alterations related to aging are defined by multiple physical facets, including changes in gross morphology, blood perfusion, glymphatic drainage, synaptic transmission, electrolyte balance, and microscopic changes in cellular metabolism [4].

Aging is correlated with an increase in the accumulation of oxidative stressors in neurons [4,5]. As one ages, there is a buildup of damaged mitochondria and reactive oxygen species (ROS) as well as a decrease in enzymatic and non-enzymatic antioxidant levels [5]. The build-up of damaged mitochondria increases oxidative damage to both mitochondrial and nuclear DNA, impairs cellular respiration and metabolism, and causes a decrease in cellular energy levels, as evidenced by reduced nicotinamide adenine dinucleotide (NAD) levels. The major ROS created in neurons include superoxide anion radicals, hydroxyl radicals, and nitric oxide. These ROS impair the function of membrane proteins required for cell metabolism. Changes in cell metabolism can manifest as decreased glucose metabolism. As ROS levels increases within a cell, key proteins responsible for maintaining adequate glucose levels in the brain become impaired [3,6].

The brain utilizes aerobic glycolysis (AG), a non-oxidative form of glycolysis, throughout childhood and adulthood [7]. Alterations in AG serve as an essential indicator for monitoring brain glucose metabolism, as other parameters, such as total brain glucose uptake, oxygen utilization, and cerebral blood flow, tend to remain too constant to detect any significant variation in normal aging [8]. Monitoring changes in AG via positron emission tomography (PET) have shown a drastic reduction in glucose metabolism, leading to the use of AG has a biomarker for normal neurological aging [7,8,9,10]. ^18^F-Fluorodeoxyglucose (FDG) positron emission tomography (PET) has been established as a leading tool in measuring changes suspected in both normal and pathological aging; ^18^F-FDG-PET/CT enables the monitoring of glucose metabolism, cerebral blood flow, and oxygen consumption [11,12]. While FDG-PET has traditionally been employed for metabolic assessment, it is worth noting that alternative tracers, such as radiowater, are also utilized in measuring blood flow and oxygen consumption [13].

Glucose consumption increases nearly linearly relative to the functional activity of resting brain regions. This allows FDG to be utilized as a tracker of functional activity in both physiological and pathological aging [12,14,15,16]. Furthermore, ^18^F-FDG-PET/CThas shown to be comparatively more sensitive in monitoring patients with moderate cognitive impairment and their progression into dementia and other neurological degenerative diseases relative to other imaging methods, including single-photon emission computed tomography (SPECT) and structural magnetic resonance imaging (MRI) [11,16]. In this study, we evaluated global and regional neurological glucose metabolism in a group of healthy individuals. Through an analysis of FDG uptake, we aimed to assist in the detection of changes that may indicate pathological aging and neurological disease.

## 2. Methods

### 2.1. Study Population

The study population comprised 73 individuals, selected from a pool of 139 subjects who participated in the Cardiovascular Molecular Calcification Assessed by 18F-Sodium Fluoride (NaF) PET/computed tomography (CT) (CAMONA) protocol between 2012 and 2016 (ClinicalTrials.gov NCT01724749) [17]. This prospective study was conducted under the purview of the Danish National Committee on Biomedical Research Ethics. All subjects gave written informed consent prior to the study.

### 2.2. Subjects

Our analysis included 73 healthy individuals (mean age: 35.8 ± 13.1 years; 17.5% male). Subjects were recruited from a random sample of Danish citizens without prior history or symptoms of cardiovascular disease. Participants with a history of pregnancy, malignancy within the past 5 years, immunodeficiency, history of deep vein thrombosis or pulmonary embolism within the prior 3 months, alcohol or illicit drug use/abuse, mental illness, and current statin therapy use were excluded from the study. Sixteen patients were excluded due to poor image quality.

### 2.3. Brain PET/CT Acquisition Protocol

All subjects underwent whole-body ^18^F-FDG-PET/CT imaging 180 min following the administration of a 4.0 MBq/kg dose of FDG. The acquisition time was 3.5 min/bed. Imaging was performed on hybrid PET/CT scanners with comparable spatial resolutions (GE Discovery RX, STE and 690/710 imaging systems; GE Healthcare, Milwaukee, WI, USA) following an overnight fast of at least 8 h and confirmation of a blood glucose concentration below 8 mmol/L. Low-dose CT imaging (140 kV, 30–110 mA, noise index 25, 0.8 s/rotation, slice thickness 3.75) was performed for attenuation correction and structural correlation with PET scans. PET scans were corrected to account for scatter, attenuation, random coincidences, and scanner dead time.

### 2.4. Brain PET/CT Data Analysis

**Figure 1:**
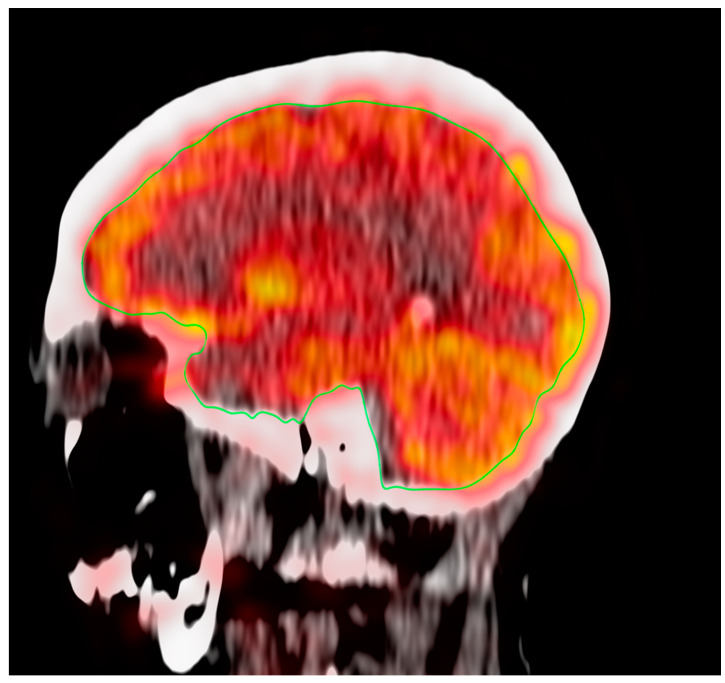
ROI for the global assessment of the supratentorial region of the brain.

OsiriX MD (Osirix MD v.13.0.1; Pixmeo, SARL, Bernex, Switzerland) was utilized to perform a global assessment of FDG uptake in the brain. A single investigator manually delineated the CT-based regions of interest (ROIs) on the fused PET/CT images for the global assessment of the supratentorial region and cerebellum. Semi-quantification of FDG uptake was calculated from the regions outlined with the hand-drawn ROIs; regions were manually traced on each sagittal slice (Figure 1). The global average standardized uptake value mean (Avg SUVmean) was measured via mapping ROS of the entire brain across the supratentorial structures and cerebellum.

**Figure 2:**
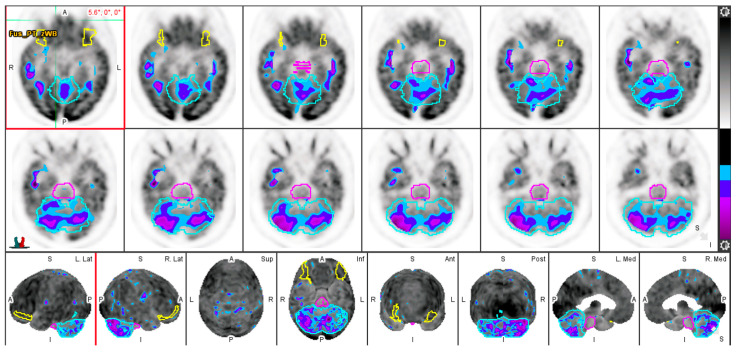
Evaluation of FDG-PET through quantitative analysis. Low FDG uptake is represented by purple and blue contours. The basis pontis (pink), cerebellum (light blue), and lateral orbital gyrus (yellow) are delineated.

To analyze FDG regional uptake in the brain, quantitative regional analysis was conducted using MIMneuro version 7.1.5 (MIM Software, Inc., Cleveland, OH, USA) through validated methods [18,19]. MIMneuro registers each brain to a standard template, allowing for increased accuracy in quantitative comparisons. MIMneuro software transposes PET data on a voxel-to-voxel basis onto a standard brain template. This template is designed for comparison with an integrated anatomical brain atlas that includes predefined regions of interest. The software then outputs normalized z-scores. For each subject, metabolic activity was normalized to their whole brain activity. The process employed linear scaling to account for individual brain size and nonlinear warping to minimize differences in brain regions between individual scans and the atlas. For each subject, metabolic activity was normalized to their whole brain activity. This program identified metabolic activity in 70 named regions included in the analysis (Figure 2).

Correlations were performed to assess associations between age and regional cerebral FDG uptake in the healthy individuals. Correlations were also performed to assess any associations between age and global cerebral FDG uptake. Pearson’s R was calculated and evaluated for significance in all variables. The threshold for significance was set at *p* < 0.05. All statistical analyses in this study were performed using R version 4.2.1 (23 June 2022).

## 3. Results

### 3.1. Clinical Characteristics of the Study Population

Table 1 provides a comprehensive overview of the demographic and clinical characteristics of the study’s participants. The study analyzed a total of 73 subjects, predominantly female (82.5%), with an average age of 35.8 ± 13.1 years and an average body mass index of 27.4 ± 4.5 kg/m^2^. This population had a low prevalence of comorbidities associated with cerebrovascular risk factors. Notably, no subjects had a history of previous stroke or transient ischemic attack.

Smoking history was noted in 42.5% of subjects. A family history of coronary artery disease and arterial hypertension was observed in 32.5% and 25.0% of the subjects, respectively. Other potential risk factors, such as hypercholesterolemia, atrial fibrillation, heart valve disease, and peripheral artery disease, were found in 15.0%, 7.5%, 5.0%, and 2.5% of subjects, respectively. In terms of risk assessment, the average 10-year Framingham risk was relatively low at 4.7%, with a 25–75th percentile range of 0.7–4.2%, while the mean CHADS-VASc score was 0.8%, indicating a generally low risk of cerebrovascular events in the studied population. As it pertains to glucose metabolism, the average HbA1c level was 36.4 ± 6.2 mmol/L. This value, which falls within the normal range, reflects a normoglycemic state across the cohort.

### 3.2. Effect of Aging on Global Cerebral Metabolic Activity

**Figure 3:**
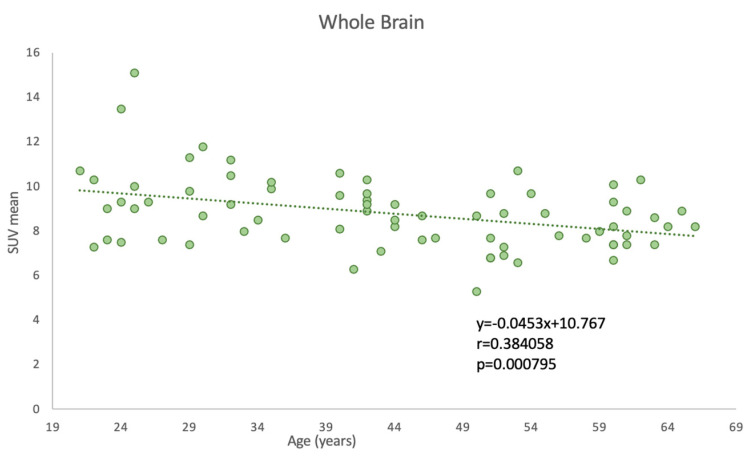
Correlation between age and PET-FDG uptake for the whole brain.

In assessing the impact of aging on whole-brain glucose metabolism, we observed a statistically significant correlation between age and global SUVmean of FDG uptake (*p* = 0.000795), indicative of whole-brain glucose metabolism. Specifically, a negative Pearson correlation coefficient of −0.384 was obtained, implying that as age increases, there is a tendency for the SUVmean FDG uptake to decrease. This finding is graphically demonstrated in Figure 3, where age is plotted against the SUVmean of the whole-brain FDG uptake.

### 3.3. Effect of Aging on Regional Cerebral Metabolic Activity

Our study identified four cerebral regions that demonstrated significant correlations between age and SUVmean FDG uptake. Region-specific analysis provides a granular view of the relationship between aging and glucose metabolism, potentially revealing region-specific aging effects. This adds another layer to our understanding of the complex process of brain aging.

**Figure 4:**
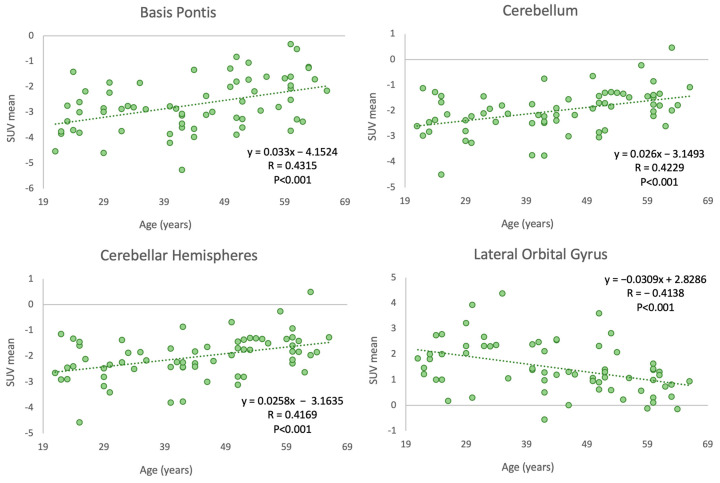
Correlation between age and PET-FDG uptake in various cerebral regions.

Among the 70 brain regions analyzed in our subjects, the basis pontis, cerebellar hemisphere, and cerebellum showed positive Pearson correlation coefficients of 0.432, 0.417, and 0.423, respectively, with *p*-values below 0.001. The lateral orbital gyrus demonstrated a negative correlation (−0.414) between age and SUVmean FDG uptake, with a *p*-value below 0.001 (Figure 4).

These region-specific correlations were further confirmed by substantial r-squared values from the ordinary least squares (OLS) linear regression analysis (R^2^ = 0.71, 0.70, 0.69, and 0.51 for the basis pontis, cerebellar hemisphere, cerebellum, and lateral orbital gyrus, respectively), suggesting that a significant proportion of the variability in glucose metabolism in these regions can be attributed to age.

## 4. Discussion

In the present study, we report a significant correlation between aging and a decrease in global brain metabolism. Our investigation underscores the profound impact of aging on whole-brain glucose metabolism. Our analysis, considering only patients without cerebrovascular risk factors, limits potential confounding effects from these factors, allowing for a more accurate estimation of the direct effect of aging on brain glucose metabolism. These findings hold substantial implications for our understanding of normal aging and are consistent with previous studies that demonstrate global cerebral metabolic activity decreases with age, primarily affecting the grey matter regions in the frontal and temporal lobe [8,16,20].

Moreover, we observed hypometabolism in the lateral orbital gyrus, a key brain region involved in emotional regulation and processing. This finding may reflect a normal change in these capacities with aging. A decrease in glucose metabolism might be associated with reduced neuronal activity in this region, potentially contributing to altered emotional processing commonly seen in elderly individuals [21,22]. Our results build upon prior conclusions from a longitudinal study by Ishibashi et al., in which decreases in FDG uptake in the anterior cingulate cortex (ACC), precuneus/posterior cingulate cortex (PC/PCC), and lateral parietal cortex (LPC) were observed over a follow-up period ranging from 4 to 11 years [23].

In our investigation, we observed three positive correlations in the basis pontis, cerebellar hemisphere, and cerebellum, which suggest an increase in glucose metabolism with advancing age. The basis pontis plays a crucial role in motor control and has been associated with sensorimotor processing [24,25]. The cerebellum and its hemispheres are also involved in motor control, as well as cognitive functions such as attention and language. Increased glucose metabolism in these regions may reflect compensatory mechanisms related to the aging process, possibly in response to degenerative changes in other parts of the brain [26,27,28]. The distinct correlation patterns observed across these different brain regions underscore the heterogeneous impact of aging on glucose metabolism within the brain, highlighting the necessity of region-specific analyses. It also raises intriguing questions about the possible physiological and clinical implications of these findings, necessitating further research to elucidate the mechanisms driving these changes and their potential impact on cognitive and emotional processing in aging individuals. Thus, these results not only enrich our understanding of brain aging and its heterogeneity but also provide a foundation for future studies that aim to dissect the complex interplay between aging, brain region functionality, and metabolic processes.

Aging is a significant risk factor for the development of cognitive neurodegenerative diseases such as Alzheimer’s Disease (AD) [29]. Previous studies have established a decline in total brain volume and cortical thinning as the main structural changes associated with pathological aging [30,31]. The key morphological aspects in aging have been referred to as cerebral atrophy, which encompasses loss of gray and white matter volume, ventricular enlargement, and sulci widening [32]. The accumulation of inclusion bodies associated with AD is observed in pathological aging; the process is linked to deficits in the glymphatic drainage system, the system responsible for clearing toxic debris and oxidative elements from the brain [33,34]. Poor blood perfusion and associated vascular diseases of the brain are also observed with both healthy and pathological aging. Impaired or reduced blood perfusion and damage to the blood–brain barrier results in hypoxic or anoxic conditions that drive the formation of oxidative stressors, causing neuronal damage and death [35,36].

Identifying structural changes can provide critical insight into defining the progression of disease and the differential vulnerabilities of brain structures to age-related pathological processes. However, anatomical changes such as regional cortical atrophy and cortical thinning can also occur in the brains of cognitively normal adults, limiting the clinical interpretability of these structural alterations [37,38].

Functional changes in neurophysiology are a central component of aging that may precede anatomical alterations [4,39,40]. ^18^F-FDG-PET/CT is, therefore, a valuable method utilized in studying functional changes in the aging brain. Through determining alterations in glucose consumption, the principal energy source of the brain, ^18^F-FDG-PET/CT can delineate the functional differences between normal aging and cognitive neurodegenerative disorders. A study by Mosconi et al. demonstrated that ^18^F-FDG-PET/CT can differentiate patients with AD from normal subjects with a sensitivity and specificity of 99% and 98%, respectively [41]. Mapping regional glucose hypometabolism can thereby provide clinicians with an objective tool in the diagnosis of dementia. In addition to its use as a differentiator between healthy and pathological aging, measuring glucose hypometabolism in cognitively normal patients with ^18^F-FDG-PET/CT may serve as a predictive marker of neurodegenerative disorders [38,42].

To our knowledge, our investigation is the first to utilize a quantitative regional analysis approach to assess the effect of aging on cerebral glucose metabolism. There are also limitations. Despite the high-resolution analysis that sets our study apart, we only utilized ^18^F-FDG-PET/CT for metabolic measurement. The utility of ^18^F-FDG-PET/CT is restricted by inherent constraints, notably its suboptimal temporal resolution and the dependency on a stable blood glucose level for accurate results. Furthermore, our study did not account for potential sex differences that could affect regional glucose metabolism across age groups, thereby limiting the generalizability of our findings. Further research is warranted to elucidate the potential effect of sex on global and regional cerebral glucose uptake. Additionally, our study did not incorporate comparative data from other advanced imaging modalities such as MRI, which could potentially offer a more comprehensive perspective and superior resolution. It should be noted that the data were collected prior to 2016, which may affect the technological robustness of the findings. PET image acquisition was not performed at the 30 to 60 min window currently recommended by the most recent guidelines; timing was in accordance with the guidelines and best practices available at the time of data collection. Lastly, our investigation did not include volumetric analysis, which may offer deeper insights into the neurological changes that occur in healthy aging.

Both anatomical changes and functional decline in glucose metabolism can present in cognitively normal adults; early detection can guide clinical treatment planning and motivate preventative measures against more severe neurogenerative disorders. Further studies with ^18^F-FDG-PET/CT are necessary in order to identify and validate the highly specific patterns of glucose hypometabolism that underlie the changes from normal to pathological aging.

## 5. Conclusions

The results of the present study demonstrate that global brain metabolism, as measured by the uptake of FDG, decreases significantly with advancing age. Regional analyses demonstrate glucose hypometabolism in the lateral orbital gyrus and hypermetabolism in the basis pontis, cerebellar hemisphere, and cerebellum. These findings collectively support the clinical utility of ^18^F-FDG-PET/CT in defining key functional differences between normal aging and cognitive neurodegenerative disease. The ability to monitor the progression of comorbid factors with glucose metabolism may help in predicting the progression of brain aging in healthy individuals as well as their risk of pathological brain aging.

## Figures and Tables

**Table 1:** Characteristics of Patients in Study (N = 73).

**Demographics**	
Female, *n* (%)	33 (82.5)
Age, years	35.8 ± 13.1
Body mass index, kg/m^2^	27.4 ± 4.5
**Comorbidities**	
Smoking history, *n* (%)	17 (42.5)
Family history of coronary artery disease, *n* (%)	13 (32.5)
Arterial hypertension, *n* (%)	10 (25.0)
Hypercholesterolemia, *n* (%)	6 (15.0)
Atrial fibrillation, *n* (%)	3 (7.5)
Heart valve disease, *n* (%)	2 (5.0)
Peripheral artery disease, *n* (%)	1 (2.5)
History of previous stroke/transient ischemic attack, *n* (%)	0 (0.0)
**Laboratory tests**	
Total cholesterol, mmol/L	5.2 ± 0.8
HDL cholesterol, mmol/L	3.3 ± 0.8
LDL cholesterol, mmol/L	1.4 ± 0.4
Triglycerides, mmol/L	1.1 ± 0.7
HbA1c, mmol/L	36.4 ± 6.2
C-reactive protein, mg/L	2.6 ± 4.1
White blood cell count, 10^9^ cells/L	6.3 ± 2.5
Fibrinogen, μmol/L	9.6 ± 1.6
Creatinine, μmol/L	81.5 ± 13
Estimated glomerular filtration rate, mL/min/1.73 m^2^	79.1 ± 13.4
**Medications**	
Aspirin, *n* (%)	6 (15.0)
Beta blockers, *n* (%)	5 (12.5)
Angiotensin-converting enzyme inhibitors/angiotensin receptor blockers, *n* (%)	5 (12.5)
Lipid-lowering medication, *n* (%)	3 (7.5)
**Risk profile**	
10-year Framingham risk, % (25–75th percentile)	4.7 (0.7–4.2)
CHADS-VASc score, % (25–75th percentile)	0.8 (0–1)
Level of physical activity, % (25–75th percentile)	2.1 (1.3–3)

## Data Availability

The data presented in this study are available on request from the corresponding author.

## References

[1] Blinkouskaya Y., Weickenmeier J. (2021). Brain Shape Changes Associated with Cerebral Atrophy in Healthy Aging and Alzheimer’s Disease. Front. Mech. Eng..

[2] Schott J.M. (2017). The Neurology of Ageing: What Is Normal?. Pract. Neurol..

[3] Mattson M.P., Arumugam T.V. (2018). Hallmarks of Brain Aging: Adaptive and Pathological Modification by Metabolic States. Cell Metab..

[4] Lee J., Kim H.-J. (2022). Normal Aging Induces Changes in the Brain and Neurodegeneration Progress: Review of the Structural, Biochemical, Metabolic, Cellular, and Molecular Changes. Front. Aging Neurosci..

[5] Castelli V., Benedetti E., Antonosante A., Catanesi M., Pitari G., Ippoliti R., Cimini A., d’Angelo M. (2019). Neuronal Cells Rearrangement during Aging and Neurodegenerative Disease: Metabolism, Oxidative Stress and Organelles Dynamic. Front. Mol. Neurosci..

[6] Massaad C.A., Klann E. (2011). Reactive Oxygen Species in the Regulation of Synaptic Plasticity and Memory. Antioxid. Redox Signal..

[7] Goyal M.S., Hawrylycz M., Miller J.A., Snyder A.Z., Raichle M.E. (2014). Aerobic Glycolysis in the Human Brain Is Associated with Development and Neotenous Gene Expression. Cell Metab..

[8] Goyal M.S., Vlassenko A.G., Blazey T.M., Su Y., Couture L.E., Durbin T.J., Bateman R.J., Benzinger T.L.-S., Morris J.C., Raichle M.E. (2017). Loss of Brain Aerobic Glycolysis in Normal Human Aging. Cell Metab..

[9] Harada N., Nishiyama S., Satoh K., Fukumoto D., Kakiuchi T., Tsukada H. (2002). Age-Related Changes in the Striatal Dopaminergic System in the Living Brain: A Multiparametric PET Study in Conscious Monkeys. Synapse.

[10] Kumar J.S.D., Mann J.J. (2014). PET Tracers for Serotonin Receptors and Their Applications. Cent. Nerv. Syst. Agents Med. Chem..

[11] Yuan Y., Gu Z.-X., Wei W.-S. (2009). Fluorodeoxyglucose-Positron-Emission Tomography, Single-Photon Emission Tomography, and Structural MR Imaging for Prediction of Rapid Conversion to Alzheimer Disease in Patients with Mild Cognitive Impairment: A Meta-Analysis. AJNR Am. J. Neuroradiol..

[12] Kato T., Inui Y., Nakamura A., Ito K. (2016). Brain Fluorodeoxyglucose (FDG) PET in Dementia. Ageing Res. Rev..

[13] Sipilä H.T., Clark J.C., Peltola O., Teräs M. (2001). An automatic [15O]H_2_O production system for heart and brain studies. J. Label. Comp. Radiopharm..

[14] Friedland R.P., Jagust W.J., Huesman R.H., Koss E., Knittel B., Mathis C.A., Ober B.A., Mazoyer B.M., Budinger T.F. (1989). Regional Cerebral Glucose Transport and Utilization in Alzheimer’s Disease. Neurology.

[15] Jagust W.J., Seab J.P., Huesman R.H., Valk P.E., Mathis C.A., Reed B.R., Coxson P.G., Budinger T.F. (1991). Diminished Glucose Transport in Alzheimer’s Disease: Dynamic PET Studies. J. Cereb. Blood Flow. Metab..

[16] De Leon M.J., Convit A., Wolf O.T., Tarshish C.Y., DeSanti S., Rusinek H., Tsui W., Kandil E., Scherer A.J., Roche A. (2001). Prediction of Cognitive Decline in Normal Elderly Subjects with 2-[18F]Fluoro-2-Deoxy-d-Glucose/Positron-Emission Tomography (FDG/PET). Proc. Natl. Acad. Sci. USA.

[17] Blomberg B.A., de Jong P.A., Thomassen A., Lam M.G.E., Vach W., Olsen M.H., Mali W.P.T.M., Narula J., Alavi A., Høilund-Carlsen P.F. (2017). Thoracic Aorta Calcification but Not Inflammation Is Associated with Increased Cardiovascular Disease Risk: Results of the CAMONA Study. Eur. J. Nucl. Med. Mol. Imaging.

[18] Teichner E.M., You J.C., Hriso C., Wintering N.A., Zabrecky G.P., Alavi A., Bazzan A.J., Monti D.A., Newberg A.B. (2021). Alterations in Cerebral Glucose Metabolism as Measured by 18F-Fluorodeoxyglucose-PET in Patients with Persistent Postconcussion Syndrome. Nucl. Med. Commun..

[19] Partovi S., Yuh R., Pirozzi S., Lu Z., Couturier S., Grosse U., Schluchter M.D., Nelson A., Jones R., O’Donnell J.K. (2017). Diagnostic Performance of an Automated Analysis Software for the Diagnosis of Alzheimer’s Dementia with 18F FDG PET. Am. J. Nucl. Med. Mol. Imaging.

[20] Deery H.A., Di Paolo R., Moran C., Egan G.F., Jamadar S.D. (2022). Lower Brain Glucose Metabolism in Normal Ageing Is Predominantly Frontal and Temporal: A Systematic Review and Pooled Effect Size and Activation Likelihood Estimates Meta-analyses. Hum. Brain Mapp..

[21] Hirose S., Osada T., Ogawa A., Tanaka M., Wada H., Yoshizawa Y., Imai Y., Machida T., Akahane M., Shirouzu I. (2016). Lateral-Medial Dissociation in Orbitofrontal Cortex-Hypothalamus Connectivity. Front. Hum. Neurosci..

[22] Nestor P.G., Nakamura M., Niznikiewicz M., Thompson E., Levitt J.J., Choate V., Shenton M.E., McCarley R.W. (2013). In Search of the Functional Neuroanatomy of Sociality: MRI Subdivisions of Orbital Frontal Cortex and Social Cognition. Soc. Cogn. Affect. Neurosci..

[23] Ishibashi K., Onishi A., Fujiwara Y., Oda K., Ishiwata K., Ishii K. (2018). Longitudinal Effects of Aging on 18F-FDG Distribution in Cognitively Normal Elderly Individuals. Sci. Rep..

[24] Manto M., Bower J.M., Conforto A.B., Delgado-García J.M., da Guarda S.N.F., Gerwig M., Habas C., Hagura N., Ivry R.B., Mariën P. (2012). Consensus Paper: Roles of the Cerebellum in Motor Control—The Diversity of Ideas on Cerebellar Involvement in Movement. Cerebellum.

[25] Schmahmann J.D., Ko R., MacMore J. (2004). The Human Basis Pontis: Motor Syndromes and Topographic Organization. Brain.

[26] Koziol L.F., Budding D., Andreasen N., D’Arrigo S., Bulgheroni S., Imamizu H., Ito M., Manto M., Marvel C., Parker K. (2014). Consensus Paper: The Cerebellum’s Role in Movement and Cognition. Cerebellum.

[27] Glickstein M., Doron K. (2008). Cerebellum: Connections and Functions. Cerebellum.

[28] Ward N.S. (2006). Compensatory Mechanisms in the Aging Motor System. Ageing Res. Rev..

[29] Hou Y., Dan X., Babbar M., Wei Y., Hasselbalch S.G., Croteau D.L., Bohr V.A. (2019). Ageing as a Risk Factor for Neurodegenerative Disease. Nat. Rev. Neurol..

[30] Dickerson B.C., Bakkour A., Salat D.H., Feczko E., Pacheco J., Greve D.N., Grodstein F., Wright C.I., Blacker D., Rosas H.D. (2009). The Cortical Signature of Alzheimer’s Disease: Regionally Specific Cortical Thinning Relates to Symptom Severity in Very Mild to Mild AD Dementia and Is Detectable in Asymptomatic Amyloid-Positive Individuals. Cereb. Cortex.

[31] Planche V., Manjon J.V., Mansencal B., Lanuza E., Tourdias T., Catheline G., Coupé P. (2022). Structural Progression of Alzheimer’s Disease over Decades: The MRI Staging Scheme. Brain Commun..

[32] Jin K., Zhang T., Shaw M., Sachdev P., Cherbuin N. (2018). Relationship Between Sulcal Characteristics and Brain Aging. Front. Aging Neurosci..

[33] Dickstein D.L., Kabaso D., Rocher A.B., Luebke J.I., Wearne S.L., Hof P.R. (2007). Changes in the Structural Complexity of the Aged Brain. Aging Cell.

[34] Nedergaard M., Goldman S.A. (2020). Glymphatic Failure as a Final Common Pathway to Dementia. Science.

[35] Peters R. (2006). Ageing and the Brain. Postgrad. Med. J..

[36] Sweeney M.D., Kisler K., Montagne A., Toga A.W., Zlokovic B.V. (2018). The Role of Brain Vasculature in Neurodegenerative Disorders. Nat. Neurosci..

[37] Hurtz S., Woo E., Kebets V., Green A.E., Zoumalan C., Wang B., Ringman J.M., Thompson P.M., Apostolova L.G. (2014). Age Effects on Cortical Thickness in Cognitively Normal Elderly Individuals. Dement. Geriatr. Cogn. Dis. Extra.

[38] Nugent S., Castellano C.-A., Goffaux P., Whittingstall K., Lepage M., Paquet N., Bocti C., Fulop T., Cunnane S.C. (2014). Glucose Hypometabolism Is Highly Localized, but Lower Cortical Thickness and Brain Atrophy Are Widespread in Cognitively Normal Older Adults. Am. J. Physiol. Endocrinol. Metab..

[39] Chételat G., Desgranges B., Landeau B., Mézenge F., Poline J.B., de la Sayette V., Viader F., Eustache F., Baron J.-C. (2008). Direct Voxel-Based Comparison between Grey Matter Hypometabolism and Atrophy in Alzheimer’s Disease. Brain.

[40] Lee J., Burkett B.J., Min H.-K., Senjem M.L., Lundt E.S., Botha H., Graff-Radford J., Barnard L.R., Gunter J.L., Schwarz C.G. (2022). Deep Learning-Based Brain Age Prediction in Normal Aging and Dementia. Nat. Aging.

[41] Mosconi L., Tsui W.H., Herholz K., Pupi A., Drzezga A., Lucignani G., Reiman E.M., Holthoff V., Kalbe E., Sorbi S. (2008). Multicenter Standardized 18F-FDG PET Diagnosis of Mild Cognitive Impairment, Alzheimer’s Disease, and Other Dementias. J. Nucl. Med..

[42] Mosconi L., Mistur R., Switalski R., Tsui W.H., Glodzik L., Li Y., Pirraglia E., De Santi S., Reisberg B., Wisniewski T. (2009). 18F-FDG-PET/CT Changes in Brain Glucose Metabolism from Normal Cognition to Pathologically Verified Alzheimer’s Disease. Eur. J. Nucl. Med. Mol. Imaging.

